# Cross Sectional Study and Risk Factors Analysis of *Francisella tularensis* in Soil Samples in Punjab Province of Pakistan

**DOI:** 10.3389/fcimb.2019.00089

**Published:** 2019-04-05

**Authors:** Javed Muhammad, Masood Rabbani, Muhammad Zubair Shabbir, Khushi Muhammad, Muhammad Taslim Ghori, Haroon Rashid Chaudhry, Zia Ul Hassnain, Tariq Jamil, Tariq Abbas, Muhammad Hamid Chaudhry, Muhammad Haisem-ur-Rasool, Muhammad Asad Ali, Muhammad Nisar, Girish S. Kirimanjeswara, Bhushan M. Jayarao

**Affiliations:** ^1^University of Veterinary and Animal Sciences, Lahore, Pakistan; ^2^University of Swabi, Swabi, Pakistan; ^3^The Islamia University, Bahawalpur, Pakistan; ^4^Department of Epidemiology and Public Health, Cholistan University of Veterinary and Animal Sciences, Bahawalpur, Pakistan; ^5^GIS Centre, University of the Punjab, Lahore, Pakistan; ^6^The Pennsylvania State University, University Park, PA, United States

**Keywords:** *Francisella tularensis*, soil, domestic animals, Punjab province, Pakistan

## Abstract

Tularemia is an endemic zoonotic disease in many parts of the world including Asia. A cross-sectional study was conducted to determine genome-based prevalence of *Francisella tularensis* (*Ft*) in soil, assess an association between its occurrence in soil and likely predictors i.e., macro and micro-nutrients and several categorical variables, and determine seroconversion in small and large ruminants. The study included a total of 2,280 soil samples representing 456 villages in eight districts of the Punjab Province of Pakistan followed by an analysis of serum antibodies in 707 ruminants. The genome of *Ft* was detected in 3.25% (*n* = 74, 95% CI: 2.60–4.06) of soil samples. Soluble salts (OR: 1.276, 95% CI: 1.043–1.562, *p* = 0.015), Ni (OR: 2.910, 95%CI: 0.795–10.644, *p* = 0.106), Mn (OR:0.733, 95% CI:0.565–0.951, *p* = 0.019), Zn (OR: 4.922, 95% CI:0.929–26.064, *p* = 0.061) and nutrients clustered together as PC-1 (OR: 4.76, 95% CI: 2.37–9.54, *p* = 0.000) and PC-3 (OR: 0.357, 95% CI: 0.640, *p* = 0.001) were found to have a positive association for the presence of *Ft* in soil. The odds of occurrence of *Ft* DNA in soil were higher at locations close to a water source, including canals, streams or drains, [χ^2^ = 6.7, OR = 1.19, 95% CI:1.05–3.09, *p* = 0.004] as well as places where animals were present [χ^2^ = 4.09, OR = 2.06, 95% CI: 1.05–4.05, *p* = 0.02]. The seroconversion was detected in 6.22% (*n* = 44, 95% CI: 4.67–8.25) of domestic animals. An occurrence of *Ft* over a wide geographical region indicates its expansion to enzootic range, and demonstrates the need for further investigation among potential disease reservoirs and at-risk populations, such as farmers and veterinarians.

## Introduction

Tularemia is caused by the bacterium *Francisella tularensis* (*Ft*), a category A classified select agent by the Center for Disease Control and Prevention (https://www.selectagents.gov/SelectAgentsandToxinsList.html). *Francisella tularensis* (*Ft*) is a pleomorphic Gram-negative intracellular bacterium (Schulert and Allen, 2006) that has zoonotic implications across many parts of the globe (Oyston, 2008; Vogler et al., 2009). Four subspecies, namely, *tularensis, mediasiatica, holarctica, and novicida*, have been identified (Sjösted, 2005; Champion et al., 2009; Penn, 2015). The presence of *Ft subspecies holarctica* has been reported in Europe and Asia, whereas *Ft subspecies tularensis* has been reported in North America (Garaizar et al., 2006). Among the Asian countries, most of the outbreaks and cases have been reported in Turkey (607 cases in 2012), China (31 cases in 1986), and Iran (36 casesin 2013) (Esmaeili et al., 2014; Gürcan, 2014). The organism has a broad and complex host distribution that includes vertebrates, invertebrates, and environmental matrices such as soil, aerosols, and water (Kuske et al., 2006; Silvestri et al., 2016). Humans can acquire infection through inhalation, an arthropod bite, ingestion of contaminated food, or water, as well as through contact with infected tissues or fluid from animals (Oyston, 2008; Ulu-Kilic and Doganay, 2014; Silvestri et al., 2016). Individuals living in rural areas or those in close proximity to animals/disease reservoirs, especially farmers and veterinarians, are considered the most at-risk population for tularemia (Lévesque et al., 1995; Ulu-Kilic and Doganay, 2014). Though glandular, oculo-glandular, ulcero-glandular, typhoidal, and pneumonic symptoms are common in affected humans, the clinical signs and severity of disease depend on the entry route and infectivity dose (<10 CFU) (Helvaci et al., 2000; Bossi et al., 2004; Pechous et al., 2009). Just as in humans, clinical signs in animals are varied. Cats are more susceptible than dogs and remain mostly in non-clinical form however in some cases, symptoms may include fever, lymphadenopathy, anorexia, oral ulceration, hepatospleenomegaly, and dehydration (Gliatto et al., 1994; Woods et al., 1998).

Different subspecies of *Ft* have been reported worldwide with varying geographic distribution and disease potential. Occurrence of tularemia caused by the most virulent type (*F*. *tularensis:* biovar A) has been reported only from North America, while cases caused by the less virulent type (*F. holarctica*: biovar B) have been observed in Europe, North America, and Asia (Garaizar et al., 2006; Oyston, 2008; Esmaeili et al., 2014; Ulu-Kilic and Doganay, 2014). However, there is a paucity of data on the enzootic range of *Ft* in Pakistan. Since *Ft* has the potential to survive and persist in the environment for a longer period of time (SjÖstedt, 2007), we undertook a study to determine the prevalence of *Ft* in soil from eight districts of Punjab province of Pakistan followed by an evaluation of seroconversion in small and large ruminants. Besides several soil characteristics that included macro- and micronutrients, the study also examined likely risk factors that could be associated with its occurrence in soil, and therefore can contribute toward human and animal exposures. It is anticipated that the findings will be valuable to local, as well as global public health agencies for evaluating potential disease burden, identifying reservoirs, and developing strategies to prevent and control tularemia in animal and human populations.

## Materials and Methods

### Study and Sampling Design

A cross-sectional study was conducted in Punjab province (31.1704°N and 72.7097°E) from 2011 to 2015. The province has nine administrative divisions, 36 districts and approximate 4,883 villages. It dominates agriculture, and has the largest human and livestock populations in the country. Punjab province contains five rivers (“punj” means five and “ab” means water) which together provide one of the country's largest irrigation systems for agricultural cultivation. Besides rivers and canals, ground-water (tube-well) as well as natural rain (barani) are being used to irrigate some of the areas in the province. Though mechanical (automatic) plowing is widespread across many districts in the province, animal-based plowing (manual) is also employed at some places in the province. We used three-stage sampling design. Since an incidence rate of *Ft* in Pakistan is not known, we selected districts representing the main livestock production areas of the province where there exists an increased annual incidence of human and animal disease (Directorates of Human and Animal Health, Punjab Province).

Assuming 50% prevalence, 95% CI and 5% margin of error, the required number of villages was 357, however, we included 456 villages representing 10% of each of the study district to increase the validity of the results using WinEpi software (http://www.winepi.net/uk/sample/indice.htm). From each village, we conveniently selected five sites for soil sampling; four were from livestock barns where human and animals were living in close proximity, while one represented an agricultural land only. The geographical coordinates were noted using Garmin (Dakota, U.S.A). After removing 3–5 inches of top-surface soil at each site, a total of 2,280 sample (~250–300 gram each) were collected from 456 villages representing districts Sheikhupura (*n* = 295), Gujranwala (*n* = 360), Faisalabad (*n* = 370), Sargodha (*n* = 370), Sahiwal (*n* = 255), D.G. Khan (*n* = 215), Chakwal (*n* = 190), and Attock (*n* = 225). A brief history of each study site along with information about different categorical variables or risk factors such as distance from animal market, main road and water source, animal's density in a village, number of households in a village, number of domestic animals in a village, cover ground (vegetation) was recorded (Table 1).

**Table 1:** Eigenvalues and percentage of intertia explained by each principal component.

**Component**	**Initial eigenvalues**			**Extraction sums of squared loadings**		
	**Total**	**% of Variance**	**Cumulative %**	**Total**	**% of Variance**	**Cumulative %**
1	2.143	30.619	30.619	2.143	30.619	30.619
2	1.914	27.339	57.957	1.914	27.339	57.957
3	1.351	19.299	77.256	1.351	19.299	77.256
4	0.895	12.783	90.039			
5	0.348	4.978	95.017			
6	0.285	4.076	99.093			
7	0.064	0.907	100.000			

### Genome Extraction and PCR Based Identification

Genomic DNA was extracted from 0.25 gram of each soil sample (PowerSoil® DNA Isolation Kit, MoBio, USA) as per manufacturer's instruction, and was subjected to real time PCR (CFX 96, BioRad, USA) using a highly sensitive and specific assay (Christensen et al., 2006) with minor modifications. The quantification of DNA was performed using the NanoDrop 1000 spectrophotometer (Thermo scientific, USA). The used real time PCR (RT-PCR) primers (Forward: 5′-CAGCATACAATAATAACCCACAAGG-3′ and Reverse: 5′-TCAGCATACTTAGTAATTGGGAAGC-3′) and probe [5′ (FAM)-TTACAATGGCAGGCTCCAGAAGGTT-3′ (TAMRA)], which targeted the tul4 gene (Lipoprotein/outer-membrane protein, 103 bp). A 25 uL reaction was comprised of a final concentration of 1X PCR buffer, 5 mM of MgCl2, 0.25 mM of dNTPs, 0.25 mg/mL of bovine serum albumin, 0.6 μM of each of forward and reverse primer, 0.025 μM of probe and 0.5 U of Taq-polymerase along with soil-extracted genomic DNA (10–30 ng). Thermal cycling conditions included one cycle of 95°C for 5 min followed by 45 cycles of each of denaturation at 94°C for 5 s and annealing at 60°C for 20 s, and then one cycle of cooling at 40°C for 1 min. The assay was optimized and validated using the control (*tul4* gene PCR products) and the proficiency testing samples that were kindly provided by the Pennsylvania State University, USA. The necessary assay controls such as dsDNA PCR product (positive control) and dDiethyl-pyrocarbonate water (negative control) were used each time. To rule out any potential contamination in processing and/or false-positivity, each sample that exhibited a positive result was gel-electrophoresed (Supplementary Material), and the process described above was repeated thrice beginning from the genome extraction.

### Serum Analysis

Blood samples (~5 mL) were collected conveniently from *Ft*-positive site representing goat (*n* = 200), sheep (*n* = 175), cattle (*n* = 179), and buffalo (*n* = 153) representing district Chakwal, Gujranwala, Faisalabad, Attock, Sahiwal, Sargodha, and Dera Ghazi Khan in Punjab Province, Pakistan. The separated sera were stored at −80°C until further use. Sera (1 μL) were analyzed for anti-*Ft*-enzyme linked immunosorbent assay (ELISA) antibodies using an SERION ELISA classic *Ft* kit (Virion/Serion, Germany) according to the manufacturer's instructions. A specific secondary alkaline phosphatase antibody (ThermoFisher, USA) was used for goat (F (ab') 2-rabbit anti-goat IgG H+L), sheep (F(ab')2-donkey anti-sheep IgG H+L), cattle (goat anti-bovine IgG (H+L), and buffalo (goat anti-bovine IgG H+L). Optical density of sera were read within 60 min at 405 nm against substrate blank and 655 for reference and field samples as per manufacturer's recommendations. Only valid samples with OD value of substrate blank < 0.25 and variation between OD values of standard serums not higher than 20% were considered for further analysis. The positive and negative serum samples were analyzed using Microsoft® Excel-based software tool SERION activity with reference to lower cut off value 0.42 and upper cut off value 1.43 provided by manufacturer. Samples below the lower cut off value were considered as negative while samples above the upper cut off value were measured as positive.

### Soil Chemistry Analysis and Risk Factors

Soil samples (~500 g) were analyzed, using previously optimized protocols for pH (Committee et al., 1978), moisture (McLean, 1982), texture (Robert and Frederick, 1995), total soluble salts (Magistad et al., 1945), phosphorus (Brown, 1998), copper, chromium, calcium, nickel, manganese, iron, cobalt, lead, cadmium, sodium, magnesium, and potassium (Soltanpour and Schwab, 1977), nitrogen (Fierer et al., 2001), and organic matter (Nelson and Sommers, 1982).

### Statistical Analysis

The RT-PCR results along with numerical (soil characteristics) and categorical variables (potential risk factors) were compiled into a Microsoft Excel spreadsheet. In an earlier study (Muhammad et al., 2017), we described association between physio-chemical characteristics of soil and the presence/absence of *Ft* DNA in soil samples. In this study, we further explored these data and other potential variables to quantify factors associated with detection of bacterial DNA in soil. The data on physio-chemical characteristics of soil were not normally distributed (Shapiro-Wilk test, *p* ≤ 0.05); therefore, Mann-Whitney *U*-test was applied to assess the effect of those numeric variables on soil positivity. In Figure 2 the variables with *p* ≥ 0.2 were included in further analyses. The correlation matrix plot revealed collinearity among the selected variables, and correlated variables (*r* ≥ 0.3) were subjected to principal component analysis (PCA) (Abdi and Williams, 2010; Pourhoseingholi et al., 2012). PCA reduces dimensionality in the data and transforms the variables into a new set of uncorrelated variables called principal components. The Bartlett test of sphericity was significant (*p* < 0.05) and The Kaiser–Meyer–Olkin (KMO) measure of sampling adequacy was 0.582 (*p* < 0.05), indicating appropriateness of dataset for PCA. The first three principal components had an eigenvalue >1 and were used as covariates in binary logistic regression. A Chi Square test was used to evaluate association between the occurrence of *Ft* DNA in soil and categorical variables. A Fisher Exact test was used as alternative to Chi square test where any of its assumption was violated. The regression model included the presence/absence of *Ft* DNA in soil samples as a dependent variable. The independent variables in the model were moisture, P, Ni, Mn, Na, Zn, PC1, PC2, and PC3. The model also included categorical variables with *p* ≥ 0.2. A backward likelihood ratio method was used to select covariates in the regression. A *p* ≤ 0.05 was considered significant in the outcome of regression analysis (Nandi et al., 2016). The data were analyzed in R using “FactoMineR,” “factoextra,” and “corrplot” packages (Table 3).

**Table 2:** Factor-loading matrix for the phyio-chemical attributes of soil sampled identified by principal component analysis.

	**Component**		
	**1**	**2**	**3**
Silt	−0.524	−0.467	0.572
Clay	0.593		−0.567
Organic Matter	−0.515	0.820	
Cd	0.693		0.523
Cr			
Pb	0.646		0.623
N	−0.533	0.809	

**Table 3:** Outcome of logistic regression analysis showing association between presences of DNA of *Francisella tularensis* soil samples and its potential risk factors.

	**B**	**S.E**.	**Wald**	**df**	**Sig**.	**Exp(B)**	**95% C.I. for EXP(B)**	
							**Lower**	**Upper**
Soluble salts	0.244	0.103	5.606	1	0.018	1.276	1.043	1.562
Ni	1.068	0.662	2.606	1	0.106	2.910	0.795	10.644
Mn	−0.310	0.133	5.483	1	0.019	0.733	0.565	0.951
Zn	1.594	0.850	3.511	1	0.061	4.922	0.929	26.064
PC1	1.560	0.355	19.294	1	0.000	4.760	2.373	9.548
PC3	−1.030	0.298	11.934	1	0.001	0.357	0.199	0.640
Distance from animal market	0.534	0.975	9.318	1	0.05	0.57	0.30	1.07
Water source (canal/stream/drain)	−2.051	2.762	13.956	1	0.004	1.19	1.19	3.09
Animal density in a village	0.927	0.175	8.59	1	0.07	1.56	0.97	2.43
Number of households in a village	−1.823	0.690	3.489	1	0.002	0.38	0.20	0.71
Domestic animal	0.121	0.378	7.321	1	0.02	2.069	1.05	4.05
Ground cover (vegetation)	−0.781	0.782	6.812	1	0.08	1.49	0.88	2.51
Constant	−3.647	1.381	6.978	1	0.008	0.026		

## Results

### The Prevalence of *Ft* in the Soil of the Studied Districts

**Figure 1:**
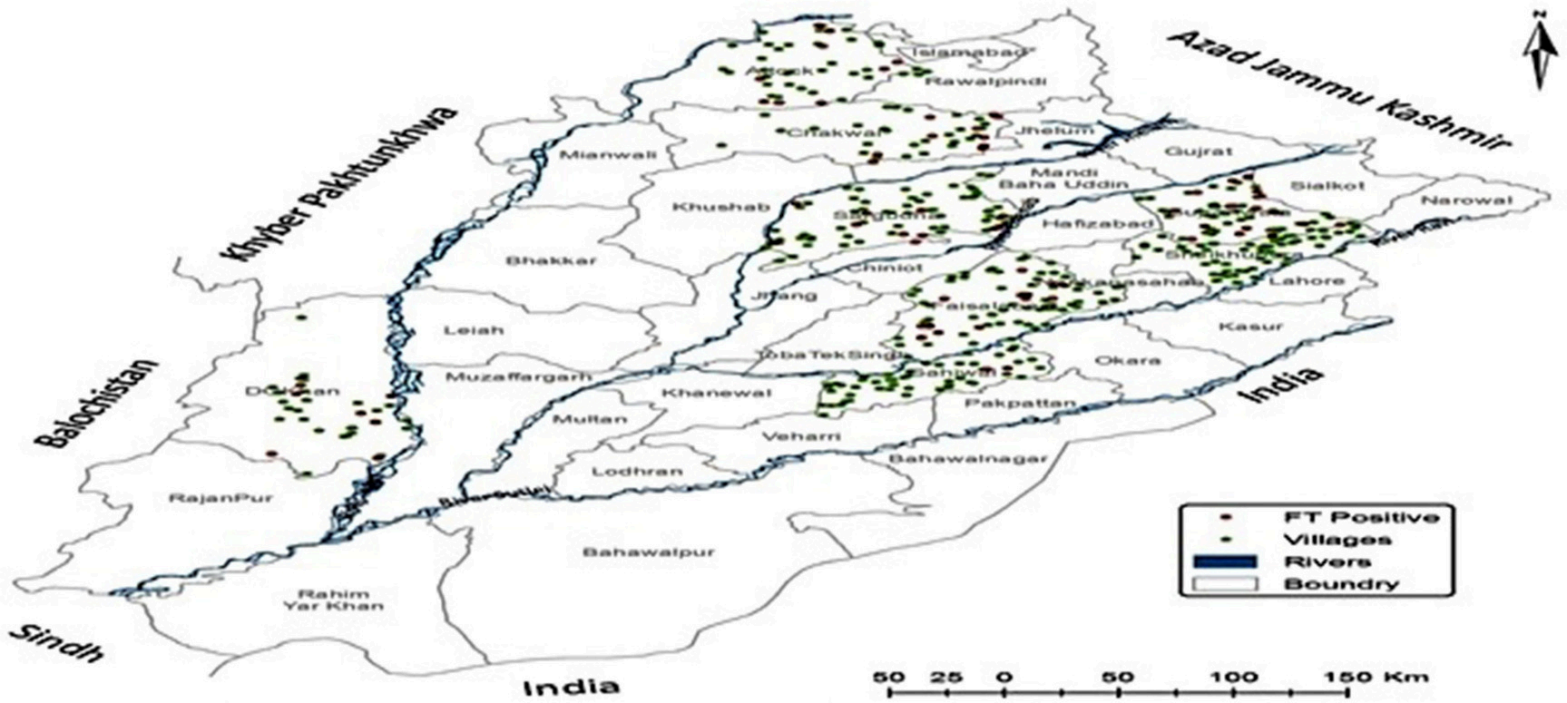
Geographical distribution of Francisella tularensis-positive soil samples in 8 districts.

Because of differences in the density of the villages in each district, the number of villages and samples varied accordingly from 38 in Chakwal (*n* = 190), to 74 in both Sargodha and Faisalabad (*n* = 370 each). *Ft* DNA was detected in 74 of 2,280 samples (3.24%, 95% CI: 2.60–4.06). There were some villages where *Ft* DNA was detected at more than one location. These included, one each from Attock (Gharibwal) and Faisalabad districts (482-GB). Compared to the other districts, an increased incidence rate of *Ft* was observed in Faisalabad (*n* = 15), Gujranwala (*n* = 17), and Attock (*n* = 10). The prevalence was highest in district Chakwal [(13.1%, (5.26%, 95% CI: 2.88–9.41) followed by Gujranwala (4.72%, 95% CI: 2.97–7.43), Attock (4.44%, 95% CI: 2.43–7.98), Faisalabad (4.05%, 95% CI: 2.47–6.58), Dera Ghazi Khan (3.72%, 95% CI: 1.90–7.17), Sargodha (3.24%, 95% CI: 1.86–5.58), and Sahiwal (0.78%, 95% CI: 0.21–2.81). None of the soil sample originating from district Sheikhupura were positive for *Ft* DNA (Figure 1). Interestingly, a total of 20 samples (4.3%, 95% CI: 2.86–6.68) representing agriculture land with no apparent human interaction were also found to be positive where there was no apparent animal and human interaction.

### Relationship Between Soil Chemistry and Categorical Variables to Occurrence of *Ft* in Soil

In the earlier study, we described association between physio-chemical characteristics of soil and presence/absence of *Ft* DNA in soil samples using a simple *t*-test and chi-square (Muhammad et al., 2017). In this study, we further explored these data and other potential variables to quantify factors associated with detection of bacterial DNA in soil. Table 1 shows eigenvalues, percentage variance and cumulative percentage of variance of principal components. The first three principals had eigenvalues > 1 and cumulatively explained 77.26% of total variance (inertia) in the dataset. Varying between + 1 and −1, the values (≥ 0.4) of the loadings represent the correlation between each variable and a principal component (Table 2). As the absolute value of the loading increases, the importance of the variable to the principal component becomes greater. Component 1 (PC1) explained 30.619% variance. It loaded positively on clay, Cd, Pb and negatively on slit, organic matter, and N. Component 2 (PC2) explained 27.339 % variance. It loaded positively on organic matter and N and negatively on silt. Component 3 (PC3) explained 19.299% variance and loaded positively on silt, Cd, Pb and negatively on clay. The variable *Ft* was significantly linked to PC1 and PC3 (*p* ≤ 0.05. For PC1, variable *Ft* explained 26% variance in the coordinates whereas correlation coefficient was 5% for PC3. The PC1 and PC3 have partitioned data into *Ft* positive and negative sites. Figure 3 displays quality of representation (squared cosine, cos2) of variables and individuals (sampling sites) along the first two principal components. The angles between variables show degree of correlation, and their lengths represent importance for the respective components. Clay, Cd, and Pb were evidenced to be more important for PC1. Organic matter and N were contributed greatly in PC2. Clay and slit were negatively correlated with each other. The sampling points in the periphery had better representation (cos2 values closer to 1).

**Figure 2:**
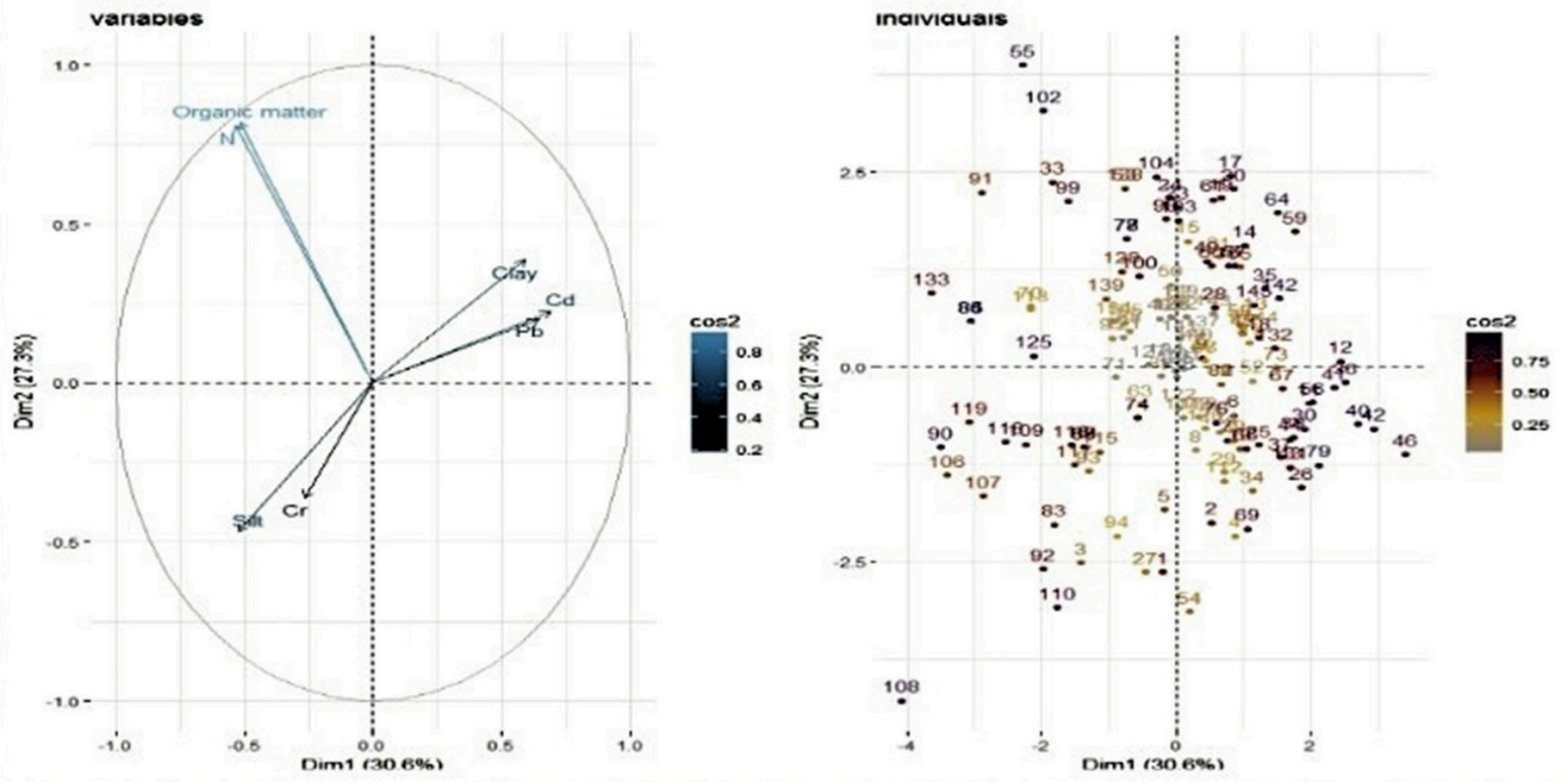
Biplot showing representation of sampling points (called individuals) and physiochemical attributes (variables) in multivariate principal component space.

**Figure 3:**
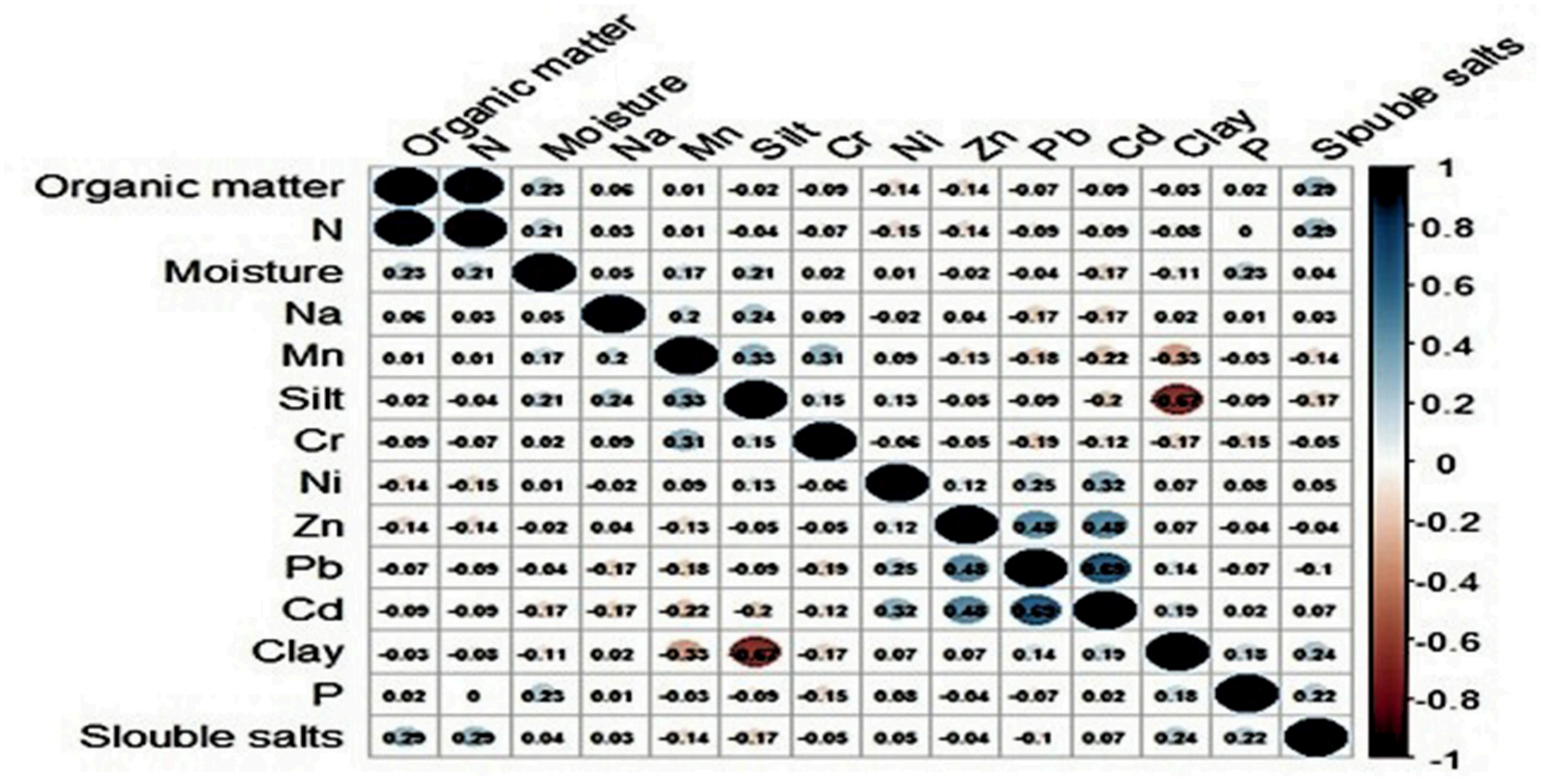
Representation (squared cosine, cos2) of variables and individuals (sampling sites) along first two principal components.

The logistic regression analysis had AIC value (143.64), Nagelkerke R Square (0.547) and −2 Log likelihood (124. 352). The accuracy of the model was 81.4. The Hosmer & Lemeshow test of the goodness of fit suggested fitness of the model at *p* = 0.792 (>0.05). Variables significantly associated with positivity of soil samples were soluble salts (OR: 1.276, 95% CI: 1.043–1.562), Ni (OR: 2.910, 95%CI: 0.795–10.644), Mn (OR:0.733, 95% CI:0.565-0.951), Zn (OR: 4.922, 95% CI:0.929–26.064), and those clustered together as PC1 (OR: 4.760, 95% CI:2.373–9.548) and PC3 (OR:0.357, 95%CI:0.199–0.640), distance from animal market (OR: 0.57, 95% CI:0.30–1.07), distance from water source (OR:1.19, 95%CI: 1.19–3.09), Animal density (OR:1.56, 95%CI: 0.97–2.43), number of households in a village (OR:0.38, 95% CI: 0.20–0.71) and domestic animals in a village (OR:2.069, 95% CI: 1.05–4.05). A unit increase in PC1 means an increase in the risk of soil positivity whereas PC3 had protective effect. Moisture, P, Na, and PC2 were non-significant and therefore removed from the regression equation through stepwise methods of covariate selection. The experimentation with regression revealed that varimax rotation and dropping some of the correlated variables did not improve the model (AIC, Nagelkerke R Square and −2 Log likelihood values not shown).

### Seroconversion in Domestic Animals to *Ft* in the Studied Districts

**Figure 4:**
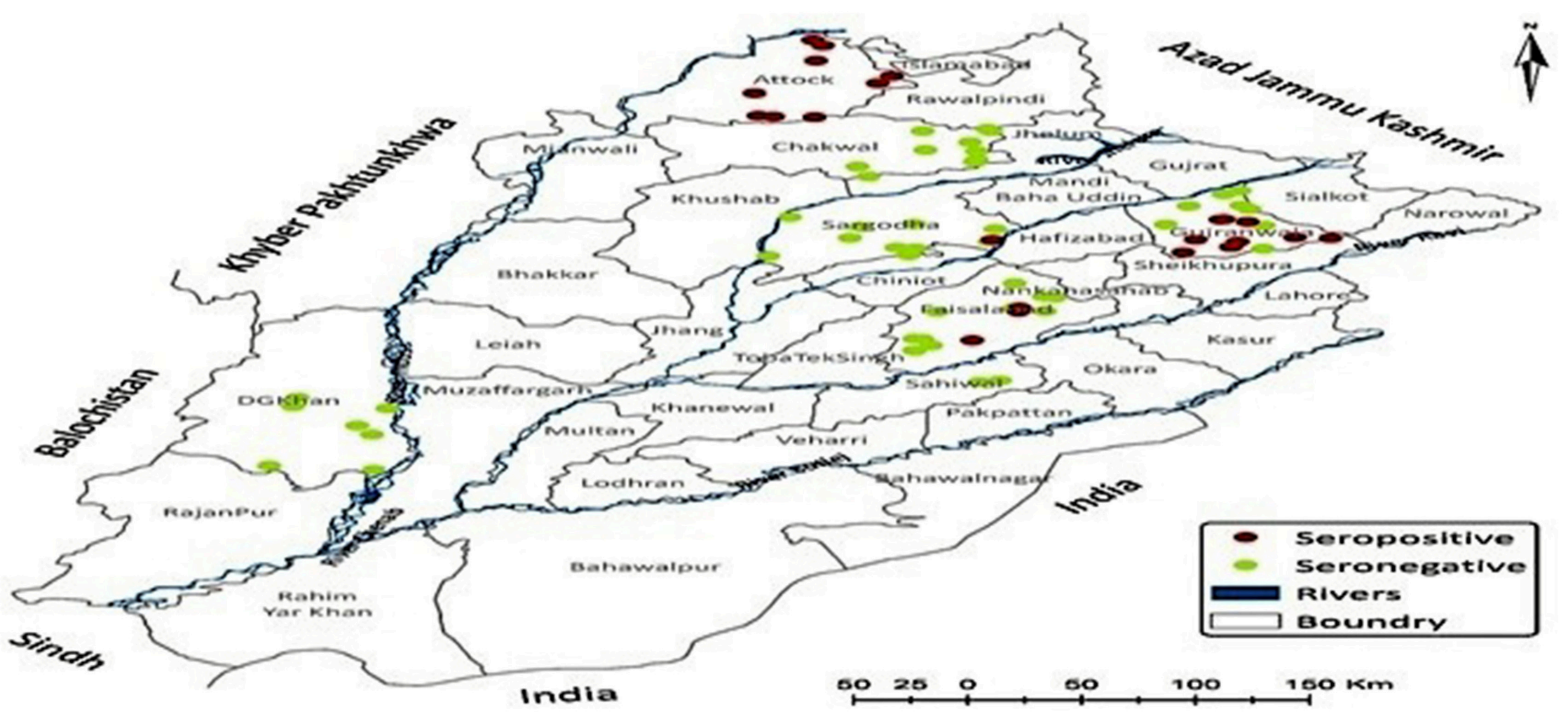
Spatial distribution of anti-Francisella tularensis seropositivity in livestock originating from study districts.

The seroconversion was found in 6.22% of small and large ruminants (*n* = 44, 95% CI: 4.67–8.25) Spatial distribution of seropositivity in animals has been illustrated in Figure 4 where a village has been red highlighted corresponding to seroconversion in any of the study animals. A significant difference (*p*<*0.05*) was found in the prevalence of serum anti-*Ft*-ELISA antibodies among cattle (11.17%, 95% CI: 7.35–16.62), and buffalo (8.49%, 95% CI: 5.04–14.0), sheep (5.7%, 95%CI: 3.13–10.19), and goat (0.5%, 95% CI: 0.00–2.78). The exposure rate was much more in large ruminants (9.94%, 95% CI: 7.17–13.63) than small ruminants (2.93%, 95 CI: 1.64–5.17). Likewise, a significant difference was observed for gender (χ^2^ = 15.35, *p* = 0.000) where seropositivity was much more in female animals (16.3%) than male animals (6.2%) while a non-significant difference was observed in age groups of small [<2 years (4%) v/s 2–4 years (2 %) v/s >4 years (14.2%), χ^2^ = 3.81, *p* = 0.14] and large ruminants [<3 years (10.8%) v/s 3–6 years (7.9%) v/s >6 years (33%), χ^2^ = 1.85 *p* = 0.39].

## Discussion

The molecular diagnostic assay (RT-PCR) had a high sensitivity and specificity with a detection limit as low as < 100 genome copies, and therefore allows simultaneous examination of numerous samples with rapid turnaround time (Christensen et al., 2006). Such assays are typically used for surveillance of extremely dangerous pathogens (EDPs) in the natural environment, particularly in settings such as Pakistan that lack a highly contained laboratory, trained personnel, culturing facilities, and repositories for isolating archives. With regards to detection of antibodies, the biological marker (lipopolysaccharide or LPS) employed for the detection of seroconversion does not produce any cross-reaction with any component of currently known bacteria (Schmitt et al., 2005; Jenzora et al., 2008), and hence is a suitable macromolecule for the detection of antibodies while monitoring a large number of samples originating from domestic animals and humans (Sharma et al., 2013).

Despite evidence of *Ft* in soil, there have been no reported tularemia outbreaks or cases in Punjab province to-date. There are a number of potential reasons for why this may be the case. Firstly, clinical cases may remain undiagnosed or misdiagnosed due to lack of laboratory based diagnostic capabilities throughout the country, as well as due to the fact that the clinical manifestations of tularemia can be fairly general. Moreover, strains isolated from the northern hemisphere are much more virulent than those isolated from Europe and Asia (SjÖstedt, 2007; Oyston, 2008). Lastly, the lack of reported cases could be due to climate and micro- and macro-nutrient characteristics of the soil supporting the persistence of *Ft* in the environment without any concomitant infection or outbreak (Dennis et al., 2001; Oyston, 2008; Alkhuder et al., 2010). Though it requires further evaluation of potential disease reservoirs (primarily rodents), and isolation and subsequent genomic characterization of prevailing strains, the more frequent occurrence of *Ft* in soil at sites close to water sources may be indicative of the prevalence of a less virulent strain (Ulu-Kilic and Doganay, 2014). Previous studies have shown that *Ft* can survive in water, soil, mud, animal waste, and frozen meat for an extended period of time (Eliasson et al., 2006; SjÖstedt, 2007). An area in close proximity to water may have a higher probability for the presence of *Ft*, however the presence of mammalian host may be required for manifestation of disease (Desvars et al., 2015). Although an increased persistence of *Ft* has been observed in environments lacking humidity (Wilkinson, 1966; Dennis et al., 2001), the organism cannot remain viable under desiccation conditions (Faith et al., 2012). A significant association between the occurrence of *Ft* in soil and the presence of animals at the same site may correspond to the presence of disease reservoirs at locations in Punjab province where *Ft* was identified, such as the rodents that are found frequently at livestock barns and surrounding areas. It has been observed that an increase in the human population can decrease the number of rodents in the fields and corresponding areas (Sutherst, 2004); in contrast, an increase in the animal population can lead to an increase in the rodent population and consequently an increase in the risk of *Ft* (Gürcan, 2014). Most of the positive samples were collected in irrigated and cropland areas (Chakwal, Gujranwala, and Faisalabad), that are interconnected with each other. In fact, compared to areas with forests and water, a previous study showed that *Ft* was primarily observed in croplands where rodents were found to be a common vector for *Ft* infections (Hightower et al., 2014).

Our findings show that it is difficult to define the relationship between the characteristics of the studied analytes and the persistence of *Ft* in soil. However, this study provides insight between *Ft* and its relationship with soil texture and metals requiring that further investigations are conducted in order to ascertain more definitive outcomes. Metals including iron, zinc, copper, and manganese play a key role in biochemical reactions and are present in most of the organism as constituents of different biological molecules (Hood and Skaar, 2012). Copper, magnesium, manganese, zinc, nickel, cadmium and sodium were observed to have a role in persistence of *Ft* in soil, although further investigation of these elements is needed. Indeed, some studies emphasized that survival and persistence of different organisms in soil is influenced by the soil texture (Nicholson et al., 2005). For instance, *Burkholderia pseudomallei* and *Ft* has the potential to survive for 30 months and 14 weeks, respectively, in clay soil (Thomas and Forbes-Faulkner, 1981; Cieslik et al., 2018). Generally, soil with an increase in clay support enhanced growth of organisms as compared to soil with increased concentration of sand (Locatelli et al., 2013). A potential association between *Ft* DNA and Magnesium could be correlated with its role while maintaining negative charge in LPS of outer membrane and therefore stability of organism in unfavorable conditions (Leadbetter and Poindexter, 2013; Wu et al., 2016). Similar to *Ft*, intracellular bacteria including *Salmonella enterica, Listeria monocytogenes, Brucella abortus*, and *Yersinia pestis* require zinc for intracellular survival and replication (Desrosiers et al., 2010; Corbett et al., 2012; Ma et al., 2015) Cadmium is normally toxic to bacteria however it has several mechanisms to develop resistance including (1) enzymes which make cell wall impermeable, (2) mechanism of efflux, (3) binding of metal ions, and (4) enzymes which convert toxic metals to non-toxic (Ron et al., 1992; Abbas et al., 2017). The persistence of *Ft* in cadmium high concentration soil presents a potential mechanism of resistance that need to be explored in future studies.

A large proportion of ruminants (goat, sheep, cattle, and buffalo) were found to be seropositive for anti-*Ft* antibodies. All the seropositive animals were clinically healthy and were devoid of symptoms suggestive of *Ft* infection. This is not surprising, because both active and passive forms of *Ft* have previously been evidenced in humans, rodents, and wild animals, (Wobeser et al., 2009) and therefore a varying rate (4–19%) of asymptomatic tularemia could be expected (Kiliç, 2010). Indeed, the clinical form of the infection depends on the route of entry of the pathogen into the body and the immune status of the host (Gürcan, 2014). The percent prevalence of anti-*Ft* antibodies was significantly higher in large ruminants (cattle and buffalo) than small ruminants (sheep and goat). Interestingly, previous studies have shown that cattle and sheep are comparatively resistant to *Ft* and remain asymptomatic (Mörner et al., 1988; Pfahler-Jung, 1989; Winn and Koneman, 2006). Some animals are very sensitive to tularemia, and succumb to infection soon after development of specific antibodies, whereas cattle, pigs, and sheep are more resistant, despite even having a significantly high antibody titer (Hopla, 1974; Valdes and Valdes, 2018). Animals may not show clinical signs even after years of infection, possibly owing to the presence of post-exposure cellular and humoral immunity (Bevanger et al., 1994; Ericsson et al., 1994; Magnarelli et al., 2007). A recent study revealed that both small and large ruminants, independent of their genders, are frequently exposed to soil contaminated with *Ft* supporting the findings by previous studies showing that cattle maintain some degree of *Ft* resistance (Dienst Jr, 1963; Scheel et al., 1992; Jacobs, 2002). Similarly, another study showed that both male and female mice are susceptible to *Ft* where male mice develop sever clinical signs and high mortality as compared to female (Sunagar et al., 2016). These observations may be due to differences in immune response development in male and female mice. An example of this was shown during *Streptococcus pneumonia* infections, where immune system inefficiency was noted to be higher in female mice as compared to male mice (Wiemken et al., 2014).

In conclusion, this study provides a preliminary confirmation of the presence of *Ft* in the Punjab Province of Pakistan and its potential association with several soil characteristics (macro- and micro-nutrients) at a higher statistical resolution. Future studies involving unexplored geographical areas of the country, disease reservoirs including rodents, and evaluation of the at-risk human population (the farmers and veterinarians), are needed.

## Ethics Statement

Blood samples were collected from cattle, buffalo, sheep, and goat following the guidelines of International Animal Care and Use Committee (IACUC) with prior consent of the farm's owner. All samples were analyzed after approval from the Ethical Research Board at the University of Veterinary and Animal Sciences, Lahore, Pakistan vide approval No: DR/236 dated 16th May, 2013.

## Author Contributions

BMJ, MZS, MR and KM: conceived and designed the study; JM, MZS, MH, ZUH, HRC, MTG, TJ, and MAA: sample collection and laboratory procedure across the study districts; JM, MZS, MN, TA and MHC: data analysis; JM, MZS, GSK and BMJ: manuscript write-up and necessary editing. All authors read and approved the manuscript.

### Conflict of Interest Statement

The authors declare that the research was conducted in the absence of any commercial or financial relationships that could be construed as a potential conflict of interest.

## References

[B1] Abbas S. Rafatullah M. Hossain K. Ismail N. Tajarudin H. Khalil H. A. (2017). A review on mechanism and future perspectives of cadmium-resistant bacteria. Int. J. Environ. Sci. Technol. 15, 243–262. 10.1007/s13762-017-1400-5

[B2] Abdi H. Williams L. J. (2010). Principal component analysis. Wiley Interdisciplin. Rev. Comput. Stat. 2, 433–459. 10.1002/wics.101

[B3] Alkhuder K. Meibom K. L. Dubail I. Dupuis M. Charbit A. (2010). Identification of trkH, encoding a potassium uptake protein required for *Francisella tularensis* systemic dissemination in mice. PLoS ONE 5:e8966. 10.1371/journal.pone.0008966 20126460 PMC2813290

[B4] Bevanger L. Maeland J. A. Kvan A. (1994). Comparative analysis of antibodies to *Francisella tularensis* antigens during the acute phase of tularemia and eight years later. Clin. Diagn. Lab. Immunol. 1, 238–240. 7496953 10.1128/cdli.1.2.238-240.1994 PMC368235

[B5] Bossi P. Tegnell A. Baka A. Van Loock F. Hendriks J. Werner A. (2004). Bichat guidelines for the clinical management of tularaemia and bioterrorism-related tularaemia. Euro Surveill. 9, E9–E10. 10.2807/esm.09.12.00503-en 15677845

[B6] Brown J. R. (1998). Recommended Chemical Soil Test Procedures for the North Central Region. Columbia, WA: Missouri Agricultural Experiment Station, University of Missouri.

[B7] Champion M. D. Zeng Q. Nix E. B. Nano F. E. Keim P. Kodira C. D.. (2009). Comparative genomic characterization of *Francisella tularensis* strains belonging to low and high virulence subspecies. PLoS Pathog. 5:e1000459. 10.1371/journal.ppat.1000459 19478886 PMC2682660

[B8] Christensen D. R. Hartman L. J. Loveless B. M. Frye M. S. Shipley M. A. Bridge D. L. (2006). Detection of biological threat agents by real-time PCR: comparison of assay performance on the RAPID, the LightCycler, and the Smart Cycler platforms. Clin. Chem. 52, 141–145. 10.1373/clinchem.2005.052522 16391330

[B9] Cieslik P. Knap J. Bielawska-Drozd A. (2018). *Francisella tularensis*—review. Postepy Mikrobiologii 57, 58–67. 10.1111/lam.13063 30106177

[B10] Committee CSS. Science CSS. McKeague J. (1978). Manual on Soil Sampling and Methods of Analysis. Pinawa, MB: Canadian Society of Soil Science.

[B11] Corbett D. Wang J. Schuler S. Lopez-Castejon G. Glenn S. Brough D.. (2012). Two zinc uptake systems contribute to the full virulence of Listeria monocytogenes during growth *in vitro* and *in vivo*. Infect. Immun. 80, 14–21. 10.1128/IAI.05904-11 22025520 PMC3255676

[B12] Dennis D. T. Inglesby T. V. Henderson D. A. Bartlett J. G. Ascher M. S. Eitzen E.. (2001). Tularemia as a biological weapon: medical and public health management. JAMA 285, 2763–2773. 10.1001/jama.285.21.2763 11386933

[B13] Desrosiers D. C. Bearden S. W. Mier I. Abney J. Paulley J. T. Fetherston J. D. (2010). Znu is the predominant zinc importer in Yersinia pestis during *in vitro* growth but is not essential for virulence. Infect. Immun. 78, 5163–5177. 10.1128/IAI.00732-10 20855510 PMC2981304

[B14] Desvars A. Furberg M. Hjertqvist M. Vidman L. Sjöstedt A. Rydén P.. (2015). Epidemiology and ecology of tularemia in Sweden, 1984–2012. Emerg. Infect. Dis. 21:32. 10.3201/eid2101.140916 25529978 PMC4285262

[B15] Dienst Jr F. (1963). Tularemia: a perusal of three hundred thirty-nine cases. J. Louisiana State Med. Soc. 115:114. 14027775

[B16] Eliasson H. Broman T. Forsman M. Bäck E. (2006). Tularemia: current epidemiology and disease management. Infect. Dis. Clin. North Am. 20, 289–311. 10.1016/j.idc.2006.03.002 16762740

[B17] Ericsson M. Sandström G. Sjöstedt A. Tärnvik A. (1994). Persistence of cell-mediated immunity and decline of humoral immunity to the intracellular bacterium *Francisella tularensis* 25 years after natural infection. J. Infect. Dis. 170, 110–114. 10.1093/infdis/170.1.110 8014484

[B18] Esmaeili S. Gooya M. M. Shirzadi M. R. Esfandiari B. Amiri F. B. Behzadi M. Y.. (2014). Seroepidemiological survey of tularemia among different groups in western Iran. Int. J. Infect. Dis. 18, 27–31. 10.1016/j.ijid.2013.08.013 24145011

[B19] Faith S. Smith L. K. Swatland A. Reed D. S. (2012). Growth conditions and environmental factors impact aerosolization but not virulence of *Francisella tularensis* infection in mice. Front. Cell. Infect. Microbiol. 2:126 10.3389/fcimb.2012.00126 23087911 PMC3468843

[B20] Fierer N. Schimel J. P. Cates R. G. Zou J. (2001). Influence of balsam poplar tannin fractions on carbon and nitrogen dynamics in Alaskan taiga floodplain soils. Soil Biol. Biochem. 33, 1827–1839. 10.1016/S0038-0717(01)00111-0

[B21] Garaizar J. Rementeria A. Porwollik S. (2006). DNA microarray technology: a new tool for the epidemiological typing of bacterial pathogens? FEMS Immunol. Med. Microbiol. 47, 178–189. 10.1111/j.1574-695X.2006.00081.x 16831204

[B22] Gliatto J. M. Rae J. F. McDonough P. L. Dasbach J. J. (1994). Feline tularemia on Nantucket island, Massachusetts. J. Vet. Diagn. Invest. 6, 102–105. 10.1177/104063879400600120 8011763

[B23] Gürcan S. (2014). Epidemiology of tularemia. Balkan Med. J. 31:3. 10.5152/balkanmedj.2014.13117 25207161 PMC4115998

[B24] Helvaci S. Gedikoglu S. Akalin H. Oral H. (2000). Tularemia in Bursa, Turkey: 205 cases in ten years. Eur. J. Epidemiol. 16, 271–276. 10.1023/A:1007610724801 10870943

[B25] Hightower J. Kracalik I. T. Vydayko N. Goodin D. Glass G. Blackburn J. K. (2014). Historical distribution and host-vector diversity of *Francisella tularensis*, the causative agent of tularemia, in Ukraine. Parasites Vectors 7:453. 10.1186/s13071-014-0453-2 25318562 PMC4200231

[B26] Hood M. I. Skaar E. P. (2012). Nutritional immunity: transition metals at the pathogen–host interface. Nat. Rev. Microbiol. 10:525. 10.1038/nrmicro2836 22796883 PMC3875331

[B27] Hopla C. E. (1974). The ecology of tularemia. Adv. Vet. Sci. Comp. Med. 18, 25–53. 4419176

[B28] Jacobs R. F. (2002). Francisella tularensis (Tularemia). Antimicrobial Therapy and Vaccines. New York, NY: Apple Trees Productions LLC.

[B29] Jenzora A. Jansen A. Ranisch H. Lierz M. Wichmann O. Grunow R. (2008). Seroprevalence study of *Francisella tularensis* among hunters in Germany. FEMS Immunol. Med. Microbiol. 53, 183–189. 10.1111/j.1574-695X.2008.00408.x 18462387

[B30] Kiliç S. (2010). A general overview of *Francisella tularensis* and the epidemiology of tularemia in Turkey. Flora 15, 37–58.

[B31] Kuske C. R. Barns S. M. Grow C. C. Merrill L. Dunbar J. (2006). Environmental survey for four pathogenic bacteria and closely related species using phylogenetic and functional genes. J. Forens. Sci. 51, 548–558. 10.1111/j.1556-4029.2006.00131.x 16696701

[B32] Leadbetter E. R. Poindexter J. S. (2013). Bacteria in Nature: Volume 3: Structure, Physiology, and Genetic Adaptability. Belrin; Heidelberg: Springer Science & Business Media.

[B33] Lévesque B. De Serres G. Higgins R. D'Halewyn M.-A. Artsob H. Grondin J.. (1995). Seroepidemiologic study of three zoonoses (leptospirosis, Q fever, and tularemia) among trappers in Québec, Canada. Clin. Diag. Lab. Immunol. 2, 496–498. 7583933 10.1128/cdli.2.4.496-498.1995 PMC170188

[B34] Locatelli A. Spor A. Jolivet C. Piveteau P. Hartmann A. (2013). Biotic and abiotic soil properties influence survival of Listeria monocytogenes in soil. PLoS ONE 8:e75969. 10.1371/journal.pone.0075969 24116083 PMC3792134

[B35] Ma L. Terwilliger A. Maresso A. W. (2015). Iron and zinc exploitation during bacterial pathogenesis. Metallomics 7, 1541–1554. 10.1039/C5MT00170F 26497057 PMC4836889

[B36] Magistad O. Reitemeier R. Wilcox L. (1945). Determination of soluble salts in soils. Soil Sci. 59, 65–76. 10.1097/00010694-194501000-00010

[B37] Magnarelli L. Levy S. Koski R. (2007). Detection of antibodies to *Francisella tularensis* in cats. Res. Vet. Sci. 82, 22–26. 10.1016/j.rvsc.2006.06.003 16914176

[B38] McLean E. (1982). Soil pH and Lime requirement, in Methods of Soil Analysis. Part 2. Chemical and Microbiological Properties (Methodsofsoilan2), ed Page A. L. (Madison, WI: American Society of Agronomy, Soil Science Society of America), 199–224.

[B39] Mörner T. Sandström G. Mattsson R. (1988). Comparison of serum and lung extracts for surveys of wild animals for antibodies to *Francisella tularensis* biovar palaearctica. J. Wildl. Dis. 24, 10–14. 3280838 10.7589/0090-3558-24.1.10

[B40] Muhammad J. Rabbani M. Muhammad K. Wasim M. Ahmad A. Sheikh A. (2017). Physicochemical factors affecting persistence of francisella tularensis in soil. J. Anim. Plant Sci. 27, 1047–1050.

[B41] Nandi A. Mandal A. Wilson M. Smith D. (2016). Flood hazard mapping in Jamaica using principal component analysis and logistic regression. Environ. Earth Sci. 75:465 10.1007/s12665-016-5323-0

[B42] Nelson D. Sommers L. E. (1982). Total carbon, organic carbon, and organic matter, in Methods of Soil Analysis. Part 2. Chemical and Microbiological Properties (methodsofsoilan2) (Madison, WI: American Society of Agronomy, Soil Science Society of America), 539–579.

[B43] Nicholson F. A. Groves S. J. Chambers B. J. (2005). Pathogen survival during livestock manure storage and following land application. Bioresource Technol. 96, 135–143. 10.1016/j.biortech.2004.02.030 15381209

[B44] Oyston P. C. (2008). Francisella tularensis: unravelling the secrets of an intracellular pathogen. J. Med. Microbiol. 57, 921–930. 10.1099/jmm.0.2008/000653-0 18628490

[B45] Pechous R. D. McCarthy T. R. Zahrt T. C. (2009). Working toward the future: insights into *Francisella tularensis* pathogenesis and vaccine development. Microbiol. Mol. Biol. Rev. 73, 684–711. 10.1128/MMBR.00028-09 19946137 PMC2786580

[B46] Penn R. L. (2015). Francisella tularensis (tularemia), in Mandell, Douglas, and Bennett's Principles and Practice of Infectious Diseases, 8th Edn, eds Bennett J. E. Blaser M. J. (Philadelphia, PA: Elsevier), 2590–2602.

[B47] Pfahler-Jung K. (1989). Die globale Verbreitung der Tularämie. Berlin: Kommission bei Duncker & Humblot.

[B48] Pourhoseingholi M. A. Mehrabi Y. Alavi-Majd H. Yavari P. (2012). Using latent variables in logistic regression to reduce multicollinearity, A case-control example: breast cancer risk factors. Italian J. Publ. Health 5, 65–71. 10.2427/5857

[B49] Robert G. Frederick R. (1995). Introductory Soil Science Laboratory Manual. New York, NY: Oxford University Press.

[B50] Ron E. Z. Minz D. Finkelstein N. Rosenberg E. (1992). Interactions of bacteria with cadmium, in Microorganisms to Combat Pollution, ed Rosenberg E. (Dordrecht: Springer), 37–46. 10.1007/978-94-011-1672-5_4

[B51] Scheel O. Sandvik T. Hoel T. Aasen S. (1992). [Tularemia in Norway. A clinical and epidemiological review]. Tidsskr. Nor. Laegeforen. 112, 635–637. 1557730

[B52] Schmitt P. Splettstoesser W. Porsch-Özcürümez M. Finke E.-J. Grunow R. (2005). A novel screening ELISA and a confirmatory Western blot useful for diagnosis and epidemiological studies of tularemia. Epidemiol. Infect. 133, 759–766. 10.1017/S0950268805003742 16050523 PMC2870305

[B53] Schulert G. S. Allen L. A. H. (2006). Differential infection of mononuclear phagocytes by Francisella tularensis: role of the macrophage mannose receptor. J. Leukocyte Biol. 80, 563–571. 10.1189/jlb.0306219 16816147 PMC1865506

[B54] Sharma N. Hotta A. Yamamoto Y. Fujita O. Uda A. Morikawa S.. (2013). Detection of *Francisella tularensis*-specific antibodies in patients with tularemia by a novel competitive enzyme-linked immunosorbent assay. Clin. Vacc. Immunol. 20, 9–16. 10.1128/CVI.00516-12 23114700 PMC3535769

[B55] Silvestri E. E. Perkins S. D. Rice E. W. Stone H. Schaefer F. W. (2016). Review of processing and analytical methods for *Francisella tularensis* in soil and water. Ann. Microbiol. 66, 77–89. 10.1007/s13213-015-1144-8

[B56] Sjösted A. (2005). Genus I. Francisella Dorofe'ev 1947, 176AL, in *Bergey1s Manual of Systematic Bacteriology, The Proteobacteria, Vol. 2*, ed George G. (New York, NY: Springer), 200–210.

[B57] SjÖstedt A. (2007). Tularemia: history, epidemiology, pathogen physiology, and clinical manifestations. Ann. NY. Acad. Sci. 1105, 1–29. 10.1196/annals.1409.009 17395726

[B58] Soltanpour P. A. Schwab A. P. (1977). A new soil test for simultaneous extraction of macro-and micro-nutrients in alkaline soils 1. Commun. Soil Sci. Plant Anal. 8, 195–207. 10.1080/00103627709366714

[B59] Sunagar R. Kumar S. Franz B. J. Gosselin E. J. (2016). Vaccination evokes gender-dependent protection against tularemia infection in C57BL/6Tac mice. Vaccine 34, 3396–3404. 10.1016/j.vaccine.2016.04.054 27182819 PMC4905791

[B60] Sutherst R. W. (2004). Global change and human vulnerability to vector-borne diseases. Clin. Microbiol. Rev. 17, 136–173. 10.1128/CMR.17.1.136-173.2004 14726459 PMC321469

[B61] Thomas A. Forbes-Faulkner J. (1981). Persistence of *pseudomonas Pseudomalleiin* soil. Austr. Veter. J. 57, 535–536. 10.1111/j.1751-0813.1981.tb05804.x 7342941

[B62] Ulu-Kilic A. Doganay M. (2014). An overview: tularemia and travel medicine. Travel Med. Infect. Dis. 12, 609–616. 10.1016/j.tmaid.2014.10.007 25457302

[B63] Valdes J. J. Valdes E. R. (2018). Biological agents: threat and response. Handb. Sec. Sci. 1–31. 10.1007/978-3-319-51761-2_16-1

[B64] Vogler A. J. Birdsell D. Price L. B. Bowers J. R. Beckstrom-Sternberg S. M. Auerbach R. K.. (2009). Phylogeography of Francisella tularensis: global expansion of a highly fit clone. J. Bacteriol. 191, 2474–2484. 10.1128/JB.01786-08 19251856 PMC2668398

[B65] Wiemken T. L. Carrico R. M. Klein S. L. Jonsson C. B. Peyrani P. Kelley R. R.. (2014). The effectiveness of the polysaccharide pneumococcal vaccine for the prevention of hospitalizations due to *Streptococcus pneumoniae* community-acquired pneumonia in the elderly differs between the sexes: results from the community-acquired pneumonia organization (capo) international cohort study. Vaccine. 32, 2198–2203. 10.1016/j.vaccine.2014.02.048 24613522

[B66] Wilkinson T. (1966). Survival of bacteria on metal surfaces. Appl. Microbiol. 14, 303–307. 5339359 10.1128/am.14.3.303-307.1966 PMC546698

[B67] Winn W. C. Koneman E. W. (2006). Koneman's Color Atlas and Textbook of Diagnostic Microbiology. Philadelphia, PA: Lippincott Williams & Wilkins.

[B68] Wobeser G. Campbell G. D. Dallaire A. McBurney S. (2009). Tularemia, plague, yersiniosis, and Tyzzer's disease in wild rodents and lagomorphs in Canada: a review. Can. Vet. J. 50:1251. 20190973 PMC2777287

[B69] Woods J. Crystal M. Morton R. Panciera R. (1998). Tularemia in two cats. J. Am. Vet. Med. Assoc. 212, 81–83. 9426784

[B70] Wu X. Ren G. Gunning III, W. T. Weaver D. A. Kalinoski A. L. Khuder S. A.. (2016). FmvB: a *Francisella tularensis* magnesium-responsive outer membrane protein that plays a role in virulence. PLoS ONE 11:e0160977. 10.1371/journal.pone.0160977 27513341 PMC4981453

