# Supplementary material for: Cross Sectional Study and Risk Factors Analysis of Francisella tularensis in Soil Samples in Punjab Province of Pakistan
Source: Front Cell Infect Microbiol. 2019 Apr 5;9:89. doi: 10.3389/fcimb.2019.00089 (PMC6460113; doi:10.3389/fcimb.2019.00089)
Supplement: Supplementary file 1 [file Table_1.docx]

***Supplementary Material***

**Prevalence and risk factors analysis of *Francisella tularensis* in soil samples; findings of a cross-sectional study in Punjab province of Pakistan**

Javed Muhammad^14^, Masood Rabbani^1*^, Muhammad Zubair Shabbir^1^, Khushi Muhammad^1^, Muhammad Taslim Ghori^2^, Haroon Rashid Chaudhry^2^, Zia Ul Hassnain^1^, Tariq Abbas^2^, Muhammad Hamid Chaudhry^3^, Muhammad Haisem-ur-Rasool^1^, Muhammad Asad Ali^1^, Tariq Jamil^1^, Muhammad Nisar^1^, Girish S Kirimanjeswara^5^, Bhushan M Jayarao^5^

**Affiliations:** ^1^University of Veterinary and Animal Sciences, Lahore, Pakistan; ^2^University College of Veterinary and Animal Sciences, The Islamia University, Bahawalpur, Pakistan; ^3^University of the Punjab, Lahore, Pakistan; ^5^The Pennsylvania State University, University Park, PA, USA

Corresponding author*: Dr. Masood Rabbani [mrabbani@uvas.edu.pk](mailto:mrabbani@uvas.edu.pk)

^4^University of Swabi, Anbar, Pakistan,


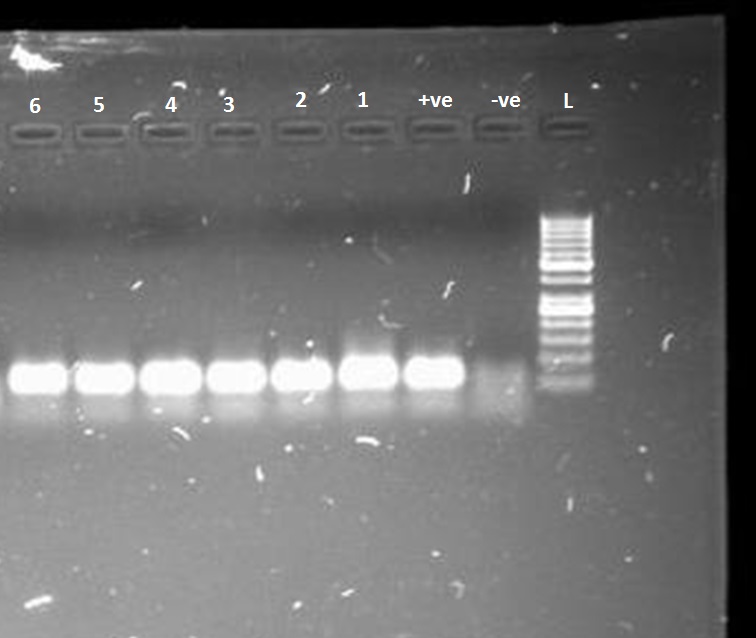


Figure 1: Gel electrophoresis of Real Time PCR products of *Francisella tularensis* tul4 gene (103 bp)

L: ladder (100 bp), -ve: Negative control, +ve: Positive control 1-6: Samples
